# Sirtuin inhibitors reduce intracellular growth of *M. tuberculosis* in human macrophages via modulation of host cell immunity

**DOI:** 10.1038/s41598-024-79136-1

**Published:** 2024-11-15

**Authors:** Sadaf Kalsum, Mira Akber, Marco Giulio Loreti, Blanka Andersson, Eva Danielson, Maria Lerm, Susanna Brighenti

**Affiliations:** 1 Center for Infectious Medicine (CIM), Department of Medicine Huddinge, Karolinska Institutet, ANA Futura, Huddinge, 141 52 Sweden; 2 Division of Medical Microbiology and Molecular Medicine, Department of Clinical and Experimental Medicine, Linköping University, Linköping, 581 83 Sweden

**Keywords:** Tuberculosis, *Mycobacterium tuberculosis*, Macrophage, Histone deacetylase (HDAC) inhibitors, Sirtuins, Immunity, Host-directed therapy, Drug discovery, Immunology, Microbiology, Medical research, Molecular medicine

## Abstract

Host-directed therapies aiming to strengthen the body’s immune system, represent an underexplored opportunity to improve treatment of tuberculosis (TB). We have previously shown in *Mycobacterium tuberculosis* (Mtb)-infection models and clinical trials that treatment with the histone deacetylase (HDAC) inhibitor, phenylbutyrate (PBA), can restore Mtb-induced impairment of antimicrobial responses and improve clinical outcomes in pulmonary TB. In this study, we evaluated the efficacy of different groups of HDAC inhibitors to reduce Mtb growth in human immune cells. A panel of 21 selected HDAC inhibitors with different specificities that are known to modulate infection or inflammation was tested using high-content live-cell imaging and analysis. Monocyte-derived macrophages or bulk peripheral blood cells (PBMCs) were infected with the green fluorescent protein (GFP)-expressing Mtb strains H37Ra or H37Rv and treated with HDAC inhibitors in the micromolar range in parallel with a combination of the first-line antibiotics, rifampicin, and isoniazid. Host cell viability in HDAC inhibitor treated cell cultures was monitored with Cytotox-red. Seven HDAC inhibitors were identified that reduced Mtb growth in macrophages > 45–75% compared to average 40% for PBA. The most effective compounds were inhibitors of the class III HDAC proteins, the sirtuins. While these compounds may exhibit their effects by improving macrophage function, one of the sirtuin inhibitors, tenovin, was also highly effective in extracellular killing of Mtb bacilli. Antimicrobial synergy testing using checkerboard assays revealed additive effects between selected sirtuin inhibitors and subinhibitory concentrations of rifampicin or isoniazid. A customized macrophage RNA array including 23 genes associated with cytokines, chemokines and inflammation, suggested that Mtb-infected macrophages are differentially modulated by the sirtuin inhibitors as compared to PBA. Altogether, these results demonstrated that sirtuin inhibitors may be further explored as promising host-directed compounds to support immune functions and reduce intracellular growth of Mtb in human cells.

**Supplementary Information:**

The online version contains supplementary material available at 10.1038/s41598-024-79136-1.

## Introduction

Tuberculosis (TB) remains the leading cause of death from an infectious disease, killing one person in the world every 20 s. Currently, over 20 clinically approved drugs are in use for TB treatment^1^. Drug-susceptible TB disease must be treated daily with a combination of at least 3–4 antibiotics for 6–9 months, and likewise the novel BPaL(M) regimen is used to treat patients with drug-resistant and multidrug-resistant (MDR-) TB disease^1^. However, more complicated MDR-TB cases will still require daily treatment with 7–9 drugs for 18–20 months and thus development of drug resistance to first- as well as second-line antibiotics is inevitable. For instance, resistance to the core drug in the BPaL(M) regimen, bedaquiline, has been identified and increased from the approval in 2012, to 3% in 2016, and 14% in 2021^2^, which threatens the effectivity of novel MDR-TB therapies and accentuates the need for alternative treatment options.

Along the search for new antibiotics, host-directed therapies (HDT) represent an underexplored opportunity to meet the challenges of treatment failure of complicated TB disease including MDR-TB^3,4^. It is well-known that the success of mycobacteria to hide and survive in alveolar macrophages and establish a persistent infection in the lung is dependent on the efficacy of the host immune system^5^. But virulent *Mycobacterium tuberculosis* (Mtb) have evolved a remarkable ability to manipulate diverse immune response pathways and undermine antimicrobial defense mechanisms including changes in host cell metabolism^6^or tissue-remodeling^7^that will compromise immune functions at the site of infection^5^. Active Mtb infection elicits a potent inflammatory response that fuel lung pathology and cavitation^7,8^, which favor TB disease progression and relapse. However, a weak immune cell response associated with immunosuppression will also allow bacterial replication and spread^5^. Accordingly, immunomodulatory compounds with the ability to restore immune defense mechanisms and/or limit immunopathology have the potential to promote cure and prevent relapse together with antibiotics^3,4^.

**Fig. 1:**
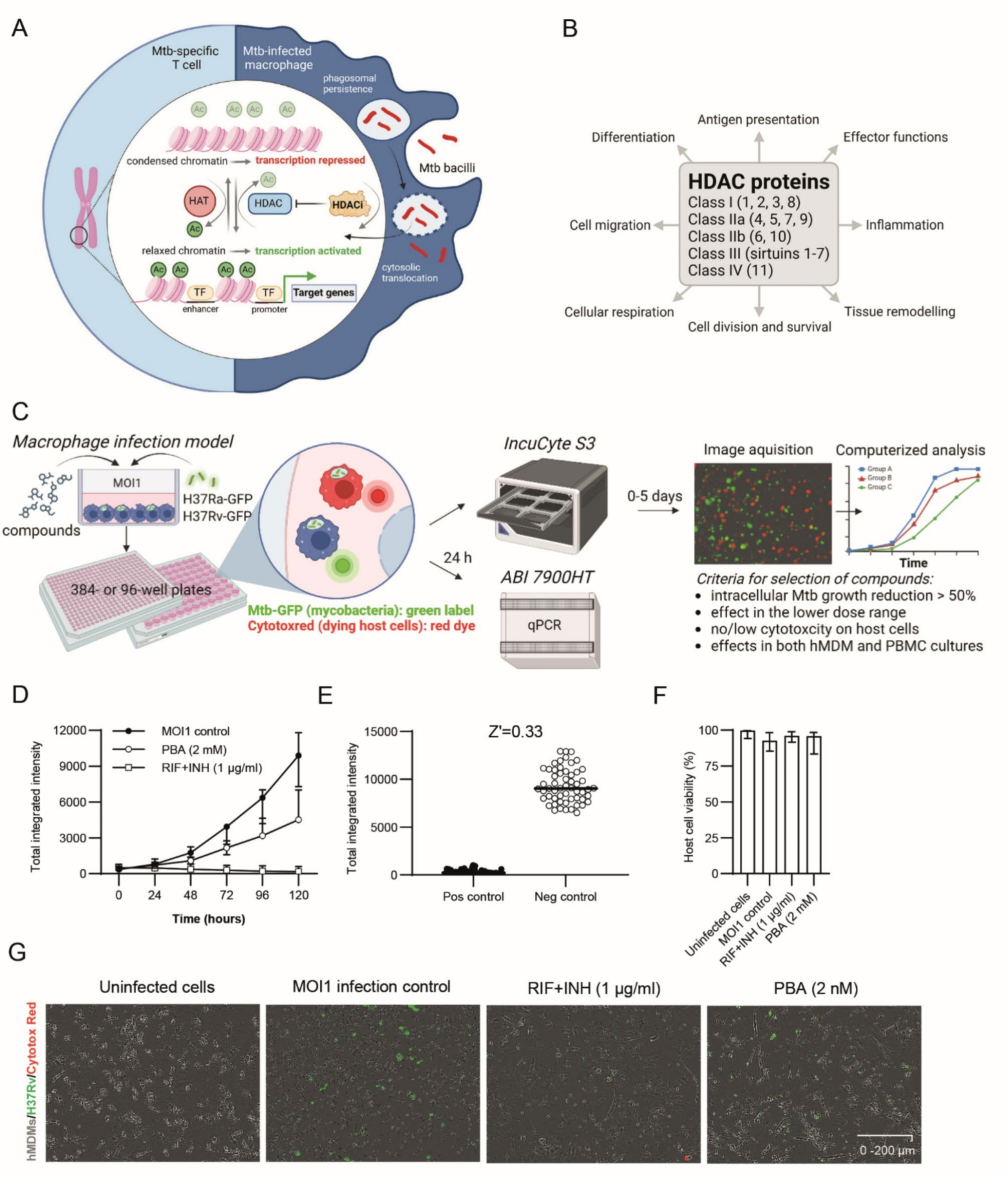
High-content imaging with IncuCyte to assess intracellular Mtb growth in human host cells. (**A**) The role of histone acetylation in transcriptional regulation of T cells (left) and Mtb-infected macrophages (right). Virulent Mtb typically induces deacetylation, chromatin compaction and inhibition of gene expression. Histone acetyltransferases (HAT), HDAC inhibitors (HDACi), acetyl group (Ac), transcription factors (TF). (**B**) Five classes containing 18 different HDAC proteins and their potential effects on cellular functions and immune cell responses. (**C**) Schematic illustration of the macrophage infection model, mycobacterial strains and experimental setup as well as data acquisition and analyses. (**D**) Longitudinal response of the Mtb infection control (MOI1), antibiotics control (RIF + INH) and internal HDAC inhibitor (PBA) control. Intracellular growth of H37Rv-GFP in hMDMs (Total integrated intensity expressed as GCU x µm2/image) was monitored in real-time (day 0–5) using IncuCyte (median +/- error). (**E**) The assay Z’ factor was determined using the mean +/- standard deviation of the negative (MOI1 infection) and positive (RIF + INH) controls. Representative data from one donor out of *n* = 3 is shown. (**F**) Viability of uninfected or H37Rv-infected hMDMs treated with RIF + INH or PBA. Data from *n* = 6 donors are presented in the bar graph (median +/- range). Host cell viability was monitored with Cytotox-red. (**G**) Representative microscopy images illustrating hMDMs (grey color) infected with H37Rv-GFP (green color) and treated with RIF + INH or PBA. Magnification x20. Illustrations in (**A**-**B**) were created with Biorender.com.

Activation of myeloid cells and T cells and their effector functions are largely controlled by epigenetic mechanisms and must undergo substantial changes in their transcriptional programs in response to Mtb^9^. It has been shown that Mtb effectively manipulates histone acetylation patterns in cells to subvert protective host immunity^10^, which typically involves histone deacetylation, chromatin compaction and inhibition of target gene expression^11^ (Fig. 1A). Accordingly, histone deacetylase (HDAC) proteins play several crucial roles in myeloid cells including Mtb-infected macrophages^12,13^, primarily through their regulation of gene expression associated with inflammatory responses, host defense mechanisms, metabolic reprogramming and macrophage polarization^14,15^ (Fig. 1B). HDAC-mediated alterations of innate immune cell function may also modulate T cell polarization and activation^16,17^. This implicates a role for HDAC inhibitors to restore immune functions in TB disease by improving macrophage and T cell responses. We have previously demonstrated that treatment with the HDAC inhibitor, phenylbutyrate (PBA), in combination with vitamin D_3_(vitD), can restore dysregulated immune cell responses in Mtb-infected macrophages by induction of the antimicrobial peptide LL-37 and autophagy, which resulted in reduced intracellular growth of both drug-susceptible and MDR-TB strains^18,19^. In clinical trials, we have observed significant effects of PBA and vitD on bacteriological and clinical outcomes in pulmonary TB^20,21^that correlated with enhanced immune functions in both myeloid cells and T cells^20^as well as reduced ER-stress^22^, which support the notion that adjunct HDT in TB can enhance clinical recovery and shorten treatment. However, PBA is a relatively weak, broad-spectrum HDAC inhibitor with activity in the mM-range^23^.

In this study, we aimed to develop our concept and search for more effective HDAC inhibitors other than PBA that can modulate innate immune responses and reduce Mtb growth in human macrophages. Therefore, selected HDAC inhibitors with documented effects in inflammation and infection or cancer^24,25^ were tested for their ability to reduce intracellular growth of Mtb in human macrophages in comparison to PBA that was used as internal HDAC inhibitor control. Phenotypic screening of compounds was performed using high-content imaging with IncuCyte. The results support a significant role of class III HDAC inhibitors, the sirtuin inhibitors, to reduce intracellular Mtb growth in human macrophages more effectively and at lower doses as compared to PBA.

## Results

**Refinement of a phenotypic assay for assessment of intracellular Mtb growth in response to selected HDAC inhibitors using high-content imaging**.

The basic principle for histone acetylation including the role of HDAC proteins and HDAC inhibitors is illustrated in Fig. 1A-B. A panel of 21 HDAC inhibitors with different specificities that are known to modulate infection or inflammation^24,25^ were selected (Table 1) and tested for the efficacy to reduce intracellular Mtb growth inside human immune cells using high-content live-cell imaging with IncuCyte (Fig. 1C). PBA, and the DNA methyltransferase (DNMT) inhibitor, decitabine, were used as comparators for intracellular killing of Mtb (Table 1). Aiming for identification of compounds with improved efficacy to reduce intracellular Mtb growth in host cells compared to PBA, pre-defined criteria for further selection of HDAC inhibitors are listed in Fig. 1C.

**Table 1:** Panel of selected HDAC inhibitors and controls.

HDAC inhibitor	Target HDAC	IC50^1^
Phenylbutyrate^2^	HDAC1, 2, 3 and 8	0.4–5.5 mM
Entinostat	HDAC1 and 3	0.51 and 1.7 µM
Apicidin	HDAC7 and 8	0.1–10 µM
Romidepsin	HDAC1 and 2	36 and 47 nM
Tubastatin A	HDAC6	15 nM
Tubacin	HDAC6	4 nM
Valproic acid	HDAC1 and 2	400 µM and 2 mM
Mocetinostat	HDAC1, 2, 3 and 11	0.15 µM
MI192	HDAC2 and 3	16 and 30 nM
Selisistat/EXS27	SIRT1	0.038 µM
AGK2	SIRT2	3.5 µM
Tenovin	SIRT1 and 2	10 and 21 µM
Suramin	SIRT1, 2 and 5	0.297 and 1.15 µM
Salermide	SIRT1 and 2	56.25 and 15.62 µM
Sirtinol	SIRT1 and 2	131 and 38 µM
Cambinol	SIRT1 and 2 (3)	56 and 59 µM
Givinostat	HDAC1 and 3	198 and 157 nM
Vorinostat/SAHA	Broad-spectrum	~ 10 nM
Belinostat	Broad-spectrum	27 nM
Panabinostat	Broad-spectrum	5 nM
Trichostatin A/TSA	Broad-spectrum	~ 1.8 nM
Dacinostat	Broad-spectrum	32 nM
Decitabine	DNMT inhibitor^3^	250–500 nM

^1^ IC50 estimated in cell-free or cell-based assays as provided by the manufacturers.

^2^ Phenylbutyrate is used as internal HDAC inhibitor control.

^3^ DNA methyltransferase (DNMT)-inhibiting cytosine nucleoside analogue.

For this work, a previously established macrophage infection model was used^19,26^ based on human monocyte-derived macrophages (hMDM) or bulk peripheral blood cells (PBMCs) that were infected with the green fluorescent protein (GFP)-expressing Mtb strains H37Ra or H37Rv and treated with HDAC inhibitors in the micromolar range in parallel with a combination of the first-line antibiotics, rifampicin (RIF) and isoniazid (INH), and also PBA (internal HDAC inhibitor control) (Fig. 1C). Initially, the Mtb infection model was optimized for longitudinal assessment of intracellular Mtb growth in primary hMDMs including titration of cell numbers, multiplicity of infection (MOI) and kinetics in response to RIF + INH and PBA (Fig. 1D). Assay Z’-factor was determined to 0.33, which is considered adequate for a phenotypic cell-based compound screening assay^27^ (Fig. 1E). The viability of the control groups at day 3 post-infection was high, and as expected only slightly reduced in Mtb-infected macrophages (Fig. 1F). Representative microscopy images obtained with the IncuCyte software demonstrated productive intracellular infection of H37Rv-GFP inside hMDMs at day 3 post-infection, which was clearly reduced after treatment with antibiotics (RIF + INH) but also PBA (Fig. 1G).

### Identification of sirtuin inhibitors as potent compounds that reduce intracellular Mtb growth in human macrophages

**Fig. 2:**
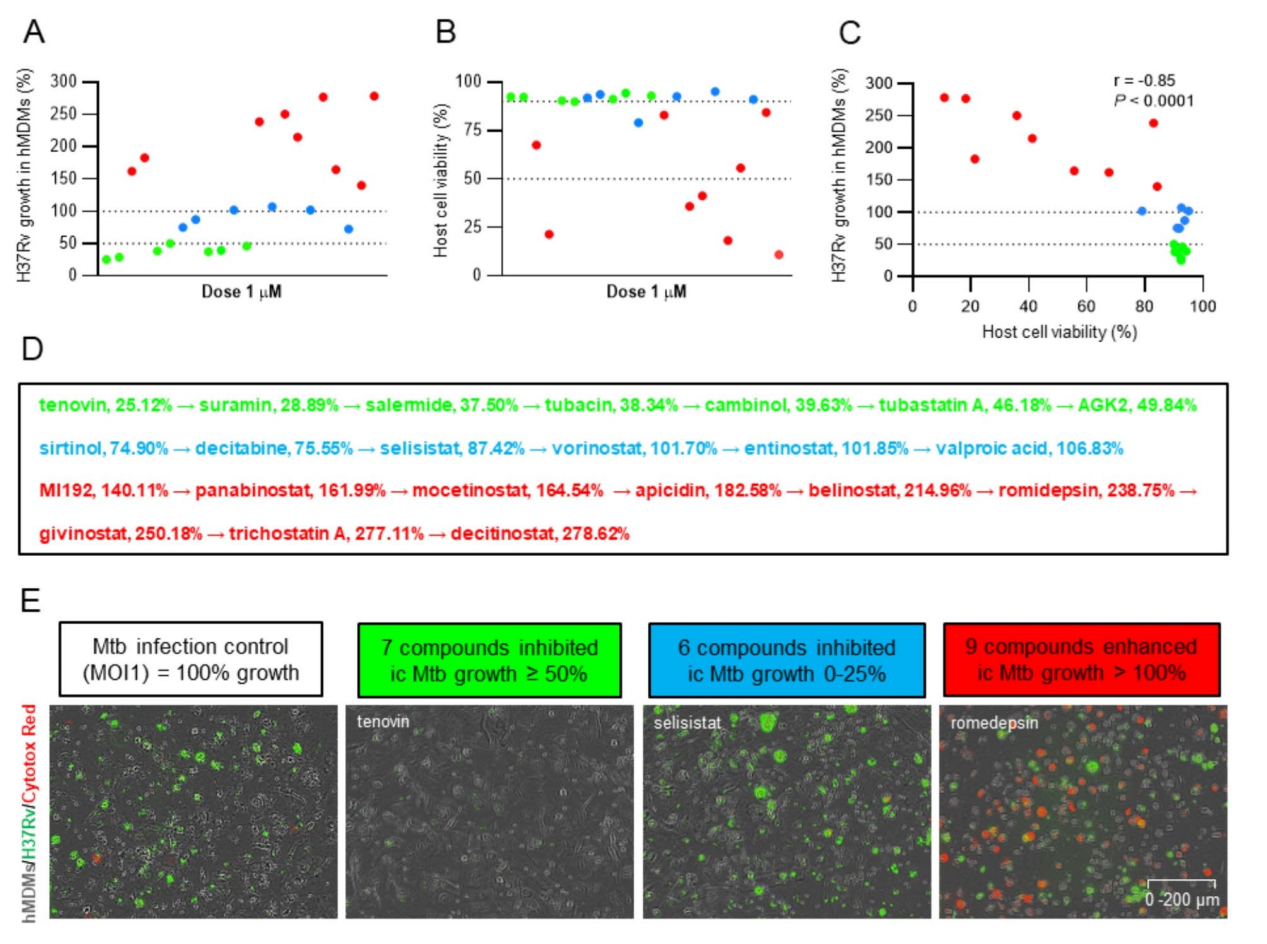
Pre-screening of HDAC inhibitor compounds on Mtb-infected macrophages. (**A**) Efficacy of the compounds to reduce intracellular growth of H37Rv inside hMDMs (%) and (**B**) assessment of macrophage viability (%). Green symbols: high efficacy (and low cytotoxicity), blue symbols: no or moderate efficacy (and low cytotoxicity, except entinostat with 79% host cell viability), and red symbols: low efficacy (and high cytotoxicity). (**C**) Correlation analysis of intracellular Mtb growth and cell viability as determined using Pearson correlation test. ****P < 0.0001. (**D**) Ranking the efficacy of the HDAC inhibitors (median Mtb growth in hMDM (%)) from the most effective (green label) to the least effective (blue and red labels) compounds. (**E**) Representative microscopy images of each condition including the MOI1 infection control, and the three different compound categories, high efficacy ≥ 50% Mtb growth inhibition (green label), moderate or low efficacy 0–25% Mtb growth inhibition (blue label), and no efficacy > 100% Mtb growth (red label). hMDMs (grey color), H37Rv bacteria (green color) and dying cells (red color) were visualized in the microscopy images. Magnification x20. Data assessed with IncuCyte at day 3 is presented in individual dot plot graphs showing the median values from *n* = 3 donors. The dotted lines in (**A** and **C**) indicate 100% and 50% intracellular Mtb growth in hMDM, respectively, and in (**B**) 90% and 50% host cell viability, respectively.

Pre-screening of the HDAC inhibitor panel (Table 1) on H37Rv-infected macrophages at 1 µM, identified three different categories consisting of compounds that effectively reduced intracellular Mtb growth, compounds that had modest or no effect on intracellular Mtb growth, and compounds with cytotoxic effects on host cell viability (Fig. 2A-C). Accordingly, seven compounds (green symbols) inhibited intracellular Mtb growth ≥ 50%, six compounds (blue symbols) had a low effect on Mtb growth 0–25%, while nine HDAC inhibitors (red symbols) enhanced intracellular Mtb growth > 100% (Fig. 2A). A lower effect of the compounds was clearly associated with enhanced cytotoxicity (Fig. 2B) as demonstrated by a significant inverse correlation (*r* = -0.85; *P* < 0.0001) between intracellular Mtb growth and host cell viability (Fig. 2C). This supports the notion that the integrity and viability of the host cell is important to restrict intracellular Mtb growth. Ranking the compounds from most to least effective in reducing Mtb growth in macrophages (Fig. 2D), demonstrated that the most potent compounds were inhibitors of the class III HDAC proteins, the sirtuins, as well as inhibitors of the HDAC6 protein. Intracellular growth of H37Rv inside hMDMs was visualized in the green channel, while dying cells were visible in red in the microscopy images (Fig. 2E).

**Fig. 3:**
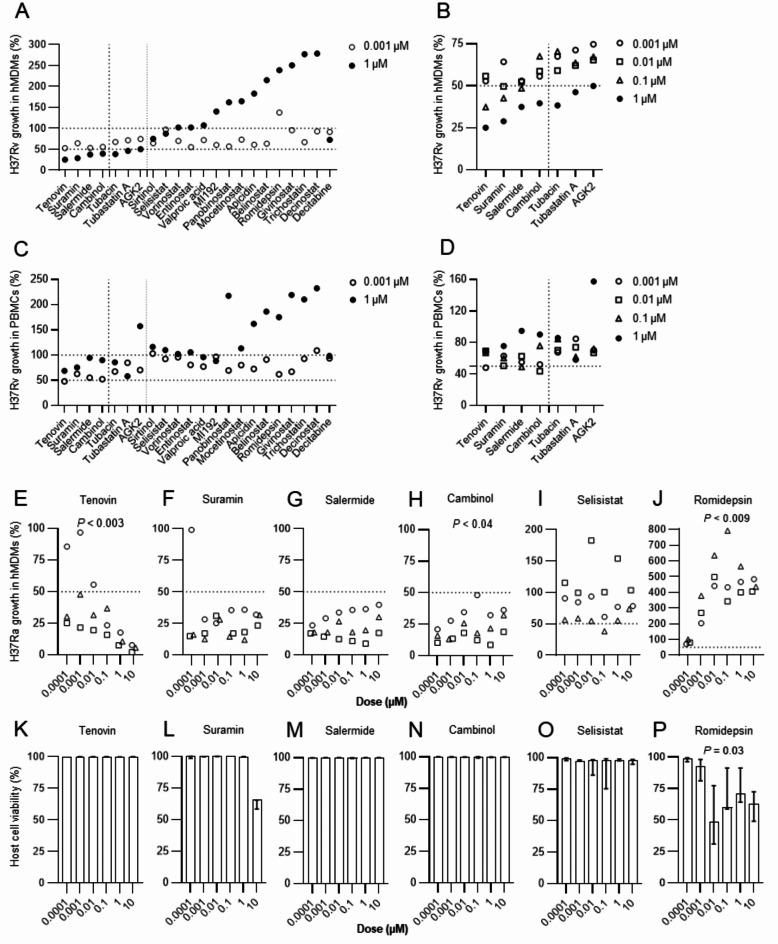
Dose-response of HDAC inhibitors assessed in Mtb-infected host cells. Intracellular growth of H37Rv-GFP inside (**A-B**) macrophages or, (**C-D**) bulk PBMCs at the indicated concentrations of HDAC inhibitor compounds. Efficacy of selected compounds to reduce intracellular growth of H37Ra-GFP inside hMDMs (%) including (**E**) tenovin, (**F**) suramin, (**G**) salermide, (**H**) cambinol, (**I**) selisistat, (**J**) romidepsin, and assessment of macrophage viability (%) including (**K**) tenovin, (**L**) suramin, (**M**) salermide, (**N**) cambinol, (**O**) selisistat, (**P**) romidepsin. Data assessed with IncuCyte at day 3 is presented in individual dot plot graphs or bar graphs (median or, median and range) from *n* = 12 (**A-D**), or *n* = 3 (**E-P**) donors. Data in (**E-P**) was analyzed using a paired Friedman test and one out of three similar experiments are shown. The horizontal dotted lines indicate 100% and 50% intracellular Mtb growth in hMDM or PBMCs, respectively, while the vertical dotted lines (**A-D**) distinguish the selected sirtuin inhibitors (thick line) as well as the seven compounds (fade line) that inhibited intracellular Mtb growth in hMDMs ≥ 50% at a concentration of 1 µM. *P < 0.05, **P < 0.01, ***P < 0.001.

Subsequently, all compounds in the test panel (Table 1) were further assessed for the ability to reduce intracellular growth of H37Rv in matched hMDMs and PBMC cultures from *n* = 12 blood donors at different concentrations (Fig. 3A-D). The results confirmed that sirtuin inhibitors were among the most potent to reduce intracellular Mtb growth, although there was a clear difference in efficacy comparing the seven top-ranked HDAC inhibitor compounds (Fig. 2A, D) to the rest of the panel (Fig. 3A, C). Whereas several HDAC inhibitors had an effect to reduce intracellular Mtb growth at lower doses (Fig. 3A, C), 10-fold dilutions ranging from 1 to 0.001 µM, suggested that four sirtuin inhibitors, tenovin, suramin, salermide and cambinol, were slightly more potent at reducing intracellular Mtb growth in both macrophages and bulk PBMCs in the lower dose range as compared to the other compounds (Fig. 3B, D). Therefore, we decided to continue experiments with a focus on these four compounds, while tubacin, tubastatin A and AGK2, were also considered effective to reduce intracellular Mtb growth in human cells.

During assay set-up and optimization, the dose response to the panel of compounds was tested using infection of hMDMs with avirulent H37Ra-GFP that can be used in biosafety level 2 (BSL-2). These experiments substantiated the findings that the four sirtuin inhibitors including tenovin, suramin, salermide, and cambinol, were most effective in reducing intracellular Mtb growth (Fig. 3E-J). Notably, at the most effective doses the compounds enhanced the efficacy of the host cells to reduce 85–98% of the avirulent mycobacterial growth (Fig. 3E-H) compared to average 75% of virulent Mtb growth (Fig. 3B) indicating that it is more difficult to kill pathogenic Mtb. Unexpectedly, only tenovin followed a typical dose-response curve with a significant dose-dependent decline of intracellular Mtb growth (*P* < 0.003) (Fig. 3E), while majority of HDAC inhibitors showed a flat or reversed dose-response (Fig. 3F-J, data not shown), which indicate that HDAC inhibitors may not follow classical pharmacodynamic dose-response relationships. Accordingly, suramin and salermide did not show a clear dose-response, while cambinol (*P* < 0.04) and romidepsin (*P* < 0.009) showed an inverse dose-response (Fig. 3F-J). However, all four sirtuin inhibitors demonstrated a significantly reduction of H37Ra growth in macrophages (*P* < 0.05 − 0.004) in comparison to the MOI1 infection control (Fig. 3E-H). Furthermore, the viability of H37Ra-infected macrophages was not much affected by the sirtuin inhibitors (Fig. 3K-N, except suramin at the 10 µM dose), while other HDAC inhibitors such as selisistat (Fig. 3O), and especially romidepsin, induced significant (*P* < 0.03) cytotoxic effects in Mtb-infected macrophages in vitro (Fig. 3P). Altogether, these data suggested that several sirtuin inhibitors effectively reduced intracellular growth of both avirulent and virulent mycobacteria in human macrophages without cytotoxic effects.

**Selected sirtuin inhibitors enhanced intracellular growth inhibition of Mtb in human host cells more effectively as compared to PBA**.

**Fig. 4:**
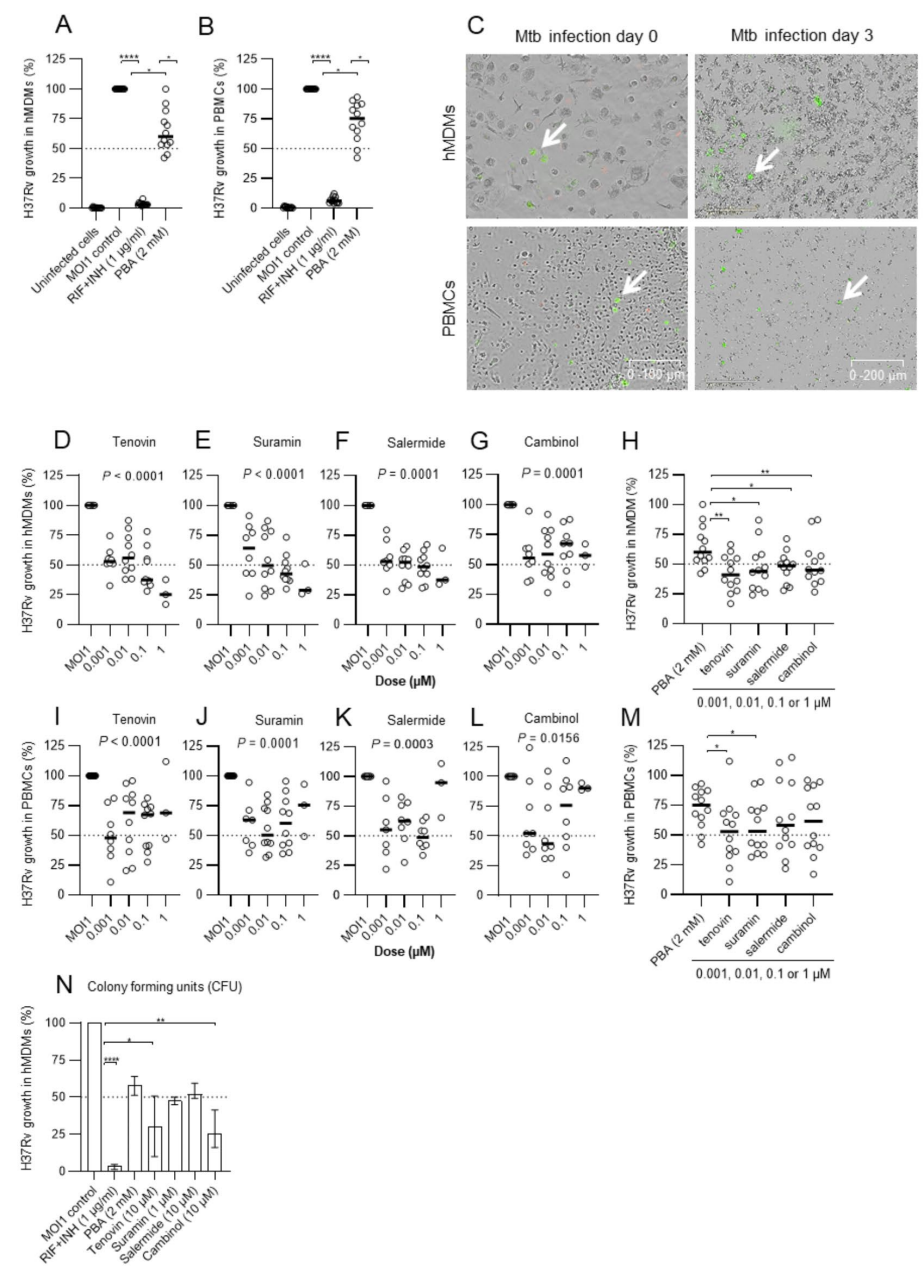
Efficacy of selected sirtuin inhibitors to reduce intracellular Mtb growth in macrophages or PBMCs. Control conditions of H37Rv-GFP growth inside (**A**) fully differentiated hMDMs, or (**B**) bulk PBMC cultures. Uninfected host cells and the MOI1 infection control was compared to the positive controls with antibiotics (RIF + INH) or PBA (internal HDAC inhibitor control) for intracellular Mtb growth (%). (**C**) Representative microscopy images of the MOI1 infection control at day 0 and 3, in hMDM (upper panel) or PBMC cultures (lower panel). Immune cells (grey color) and H37Rv bacteria (green color) were visualized in the microscopy images. Arrows indicate H37Rv-GFP infected cells. Magnification x20. Note the smaller size of the lymphocytes compared to hMDMs. Efficacy of the selected compounds, tenovin, suramin, salermide and cambinol, to reduce intracellular growth of H37Rv-GFP inside hMDMs (**D-G**) or PBMC cultures (**I-L**), including comparison of intracellular Mtb growth reduction by treatment with PBA or the sirtuin inhibitors in (**H**) hMDMs or (**M**) PBMCs. In Fig. 4H and M, respectively, individual data from *n* = 12 donors present the µM-doses for each selected sirtuin inhibitor that were most effective (0.001, 0.01, 0.1 or 1 µM) in reducing intracellular Mtb growth as compared to 2mM of PBA. (**N**) Efficacy of the selected compounds to reduce intracellular growth of H37Rv-GFP inside hMDMs assessed using CFU counts at day 3. Data assessed with IncuCyte at day 3 is presented in individual dot plot graphs or bar graphs (median and range) from *n* = 12 donors (*n* = 4 donors for CFU counts in (**N**)) and was analyzed using a paired Friedman test (**A-B**), Kruskal-Wallis and Dunn´s multiple comparisons test (**D-G** and **I-L**) and repeated measures ANOVA with uncorrected Fisher’s LSD (**H**,** M**). The dotted lines indicate 50% intracellular Mtb growth and the MOI1 control was set to 100%. *P < 0.05, **P < 0.01, ****P ≤ 0.0001.

Next, the MOI1 infection control was compared to the positive controls with antibiotics (RIF + INH) or PBA (internal HDAC inhibitor control) for intracellular Mtb growth in both fully differentiated macrophages (Fig. 4A) and bulk PBMCs (Fig. 4B) containing monocytes. Accordingly, RIF + INH or PBA reduced H37Rv growth in macrophages with 97.1% and 40.0% (Fig. 4A, median values), respectively, as compared to 93.9% and 24.9% in bulk PBMC cultures (Fig. 4B, median values), indicating that intracellular growth reduction of Mtb is mostly effective in mature macrophages regardless of treatment. There was a significant growth reduction of Mtb in both macrophages and PBMCs upon treatment with either RIF + INH (*P* < 0.0001) or PBA (*P* < 0.02 and *P* < 0.01), and there was also a significant difference between RIF + INH and the PBA control (*P* < 0.01) (Fig. 4A-B). The morphological difference illustrating large myeloid cells and smaller lymphocytes in PBMC cultures at day 0 and 3 of H37Rv infection is illustrated in Fig. 4C.

In line with pre-screening experiments and infection with H37Ra, the selected sirtuin inhibitors reduced intracellular Mtb growth in macrophages 44.4–75% (Fig. 4D-F, median values: tenovin 74.9%, suramin 71.1%, salermide 62.5%, and cambinol 44.4%), as compared to median 40.0% for PBA (Fig. 4A). Importantly, the sirtuin inhibitors exhibited their optimal effects in the µM-range compared to mM-range for PBA (Fig. 4A-K). Similarly, the sirtuin inhibitors reduced intracellular Mtb growth in bulk PBMC cultures with a max efficacy of 43.5–50.3% (Fig. 4H-FK, median values: tenovin 48.1%, suramin 50.3%, salermide 48.9%, and cambinol 43.5%) compared to median 24.9% for PBA (Fig. 4B). While 1 µM was typically the most effective dose to reduce Mtb growth in macrophages (Fig. 4D-G), a lower dose range was more beneficial to reduce Mtb growth in PBMC cultures (Fig. 4H-K). In general, the donor-to-donor variation in the response to the HDAC inhibitors tested in Table 1was large, which is consistent with our experience from previous studies^19,26^. Overall, intracellular Mtb growth reduction compared to the MOI1 infection control was significant in both macrophage (*P* ≤ 0.0001) and PBMC cultures (*P* ≤ 0.016–0.0001) (Fig. 4D-K).

As PBA was used as a global comparator for reduction of Mtb growth in host immune cells, we also performed separate analyses comparing the treatment response of the internal PBA control (2 mM) to the different sirtuin inhibitors in the µM-range (Fig. 4H and M). Mtb growth inhibition in macrophages was significantly reduced by tenovin (*P* < 0.004), suramin (*P* < 0.04), salermide (*P* < 0.008) and cambinol (*P* < 0.02) in comparison to PBA (Fig. 4H). Similarly, Mtb growth inhibition in bulk PBMC cultures was also significantly reduced by both tenovin (*P* < 0.05), and suramin (*P* < 0.03) in comparison to PBA (Fig. 4M).

Furthermore, standard CFU counts revealed that all sirtuin inhibitors could reduce intracellular Mtb growth in macrophages ranging from 25.7 to 51.9% compared to 58.1% for PBA (Fig. 4L, median values). However, the least effective sirtuin inhibitor as assessed with IncuCyte, which was cambinol, appeared to be the most effective sirtuin inhibitor assessed with CFU counts (Fig. 4G and L). Altogether, these experiments demonstrated the efficacy of several HDAC inhibitors that were more effective to reduce intracellular Mtb growth in host cells compared to PBA.

**No effect of sirtuin inhibitors on planktonic Mtb growth except tenovin that is highly effective in extracellular killing of Mtb bacilli**.

**Fig. 5:**
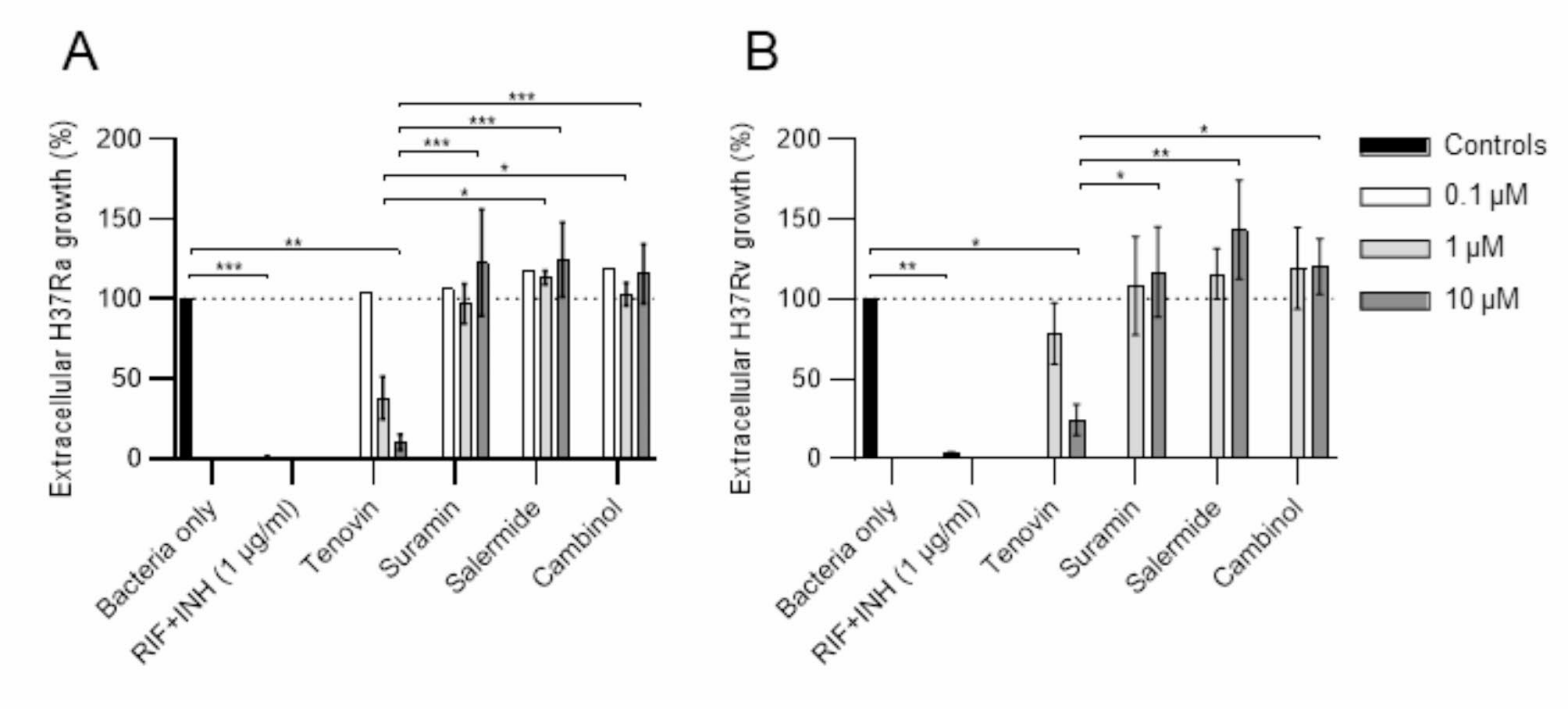
Efficacy of selected sirtuin inhibitors to reduce extracellular mycobacterial growth. (**A**) H37Ra-GFP, and (**B**) H37Rv-GFP planktonic bacterial cultures were untreated (bacteria only) or treated with the positive control (RIF + INH) or tenovin, suramin, salermide or cambinol at the indicated concentrations. Data assessed with IncuCyte at day 3 is presented in bar graphs (mean and standard error) from *n* = 3 different bacterial batches and was analyzed using a two-way RM ANOVA and Tukey´s multiple comparisons test. Selected statistical differences are shown. The dotted lines indicate 100% Mtb growth control. *P < 0.05, **P < 0.01, ***P < 0.001.

We have previously showed that PBA reduce intracellular killing of Mtb together with vitD via induction of macrophage immunity^19^. To exclude the possibility that the sirtuin inhibitors may exert direct effects on the bacteria that contributes to extracellular killing, the effects on H37Ra (Fig. 5A) or H37Rv (Fig. 5B) planktonic cultures were investigated in the absence of immune cells but in the presence of RIF + INH as well as the different sirtuin inhibitors. These experiments revealed that tenovin had a dose-dependent effect to reduce extracellular growth of both H37Ra (*P* < 0.002) (Fig. 5A) and H37Rv (*P* < 0.04) (Fig. 5B) as compared to untreated bacteria, while the other sirtuin inhibitors, suramin, salermide and cambinol, had no effect on reducing growth of extracellular Mtb bacilli (Fig. 5A-B). There was also a significant difference in extracellular H37Ra (*P* < 0.038–0.0001) and H37Rv (*P* < 0.024–0.002) growth upon treatment with tenovin compared to suramin, salermide or cambinol (Fig. 5A-B). Similar to the observations of intracellular killing of H37Ra (Fig. 3) and H37Rv (Fig. 4) in human macrophages, killing of planktonic avirulent H37Ra mycobacteria was more effective compared to killing of virulent H37Rv mycobacteria, also at lower doses of tenovin (Fig. 5A-B). Accordingly, tenovin was highly effective in killing of both intracellular (Figs. 3A and 4D, H, M and N) and extracellular Mtb (Fig. 5A-B), whereas the other sirtuin inhibitors likely reduce Mtb growth solely by improving intracellular host cell immunity.

**Additive effects on reduction of intracellular Mtb growth in macrophages upon combination treatment with subinhibitory concentrations of RIF or INH**.

**Fig. 6:**
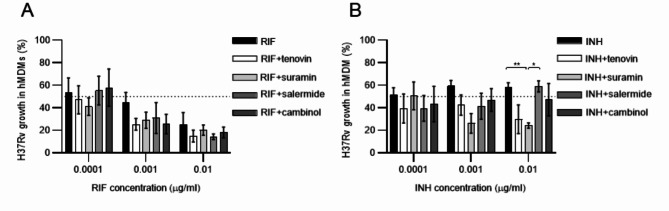
Checkerboard assay to study interactions between primary antibiotics and selected sirtuin inhibitors. hMDMs infected with H37Rv-GFP were treated with (**A**) RIF or (**B**) INH using sub-inhibitory concentrations (0.001, 0.01 and 0.1 µg/ml) in combination with a fixed dose (1 µM) of tenovin, suramin, salermide or cambinol. Data assessed with IncuCyte at day 3 is presented in bar graphs (mean and standard error) from *n* = 4 donors and was analyzed using a two-way RM ANOVA and Tukey´s multiple comparisons test. The dotted lines indicate 50% intracellular Mtb growth. *P < 0.05, **P < 0.01.

As antibiotics and host-directed compounds may use different mechanisms to reduce intracellular growth of Mtb, we next investigated the potential interaction between fixed doses (1 µM) of the sirtuin inhibitors and subinhibitory concentrations (0.001, 0.1 and 0.1 µg/ml) of RIF (Fig. 6A) and INH (Fig. 6B) using checkerboard assay. Intracellular Mtb growth was reduced by RIF in a concentration dependent manner (Fig. 6A), while the bacteriostatic effect of INH ranged from 40 to 50% using these lower doses (Fig. 6B). The relative reduction of intracellular Mtb growth by combination treatment with RIF (0.001–0.01 mg/ml) and the selected sirtuin inhibitors ranged between 20 and 45% (Fig. 6A). The effects were significant upon combination treatment with INH and suramin (*P* < 0.0068) but a relative reduction in Mtb growth was also observed in the presence of tenovin, demonstrating a 50–60% growth reduction by combining INH and suramin or tenovin (Fig. 6B). Calculation of the interaction quotients (growth index of the drug combination/growth index of RIF or INH single treatments) suggested a synergistic interaction between INH (0.01 µg/ml) and suramin (quotient < 0.5) and additive effects between INH and tenovin (quotient > 0.5 < 0.75), but also additive effects between RIF (0.001 µg/ml) and the different sirtuin inhibitors. No drug interactions were detected upon treatment with INH and salmermide or cambinol (Fig. 6B). Thus, the antimicrobial synergy testing supported synergistic or additive effects between selected sirtuin inhibitors and 10-100-fold lower concentrations of RIF and INH in reduction of intracellular Mtb growth in macrophages.

## Regulation of inflammation in Mtb-infected macrophages in response to treatment with sirtuin inhibitors

**Fig. 7:**
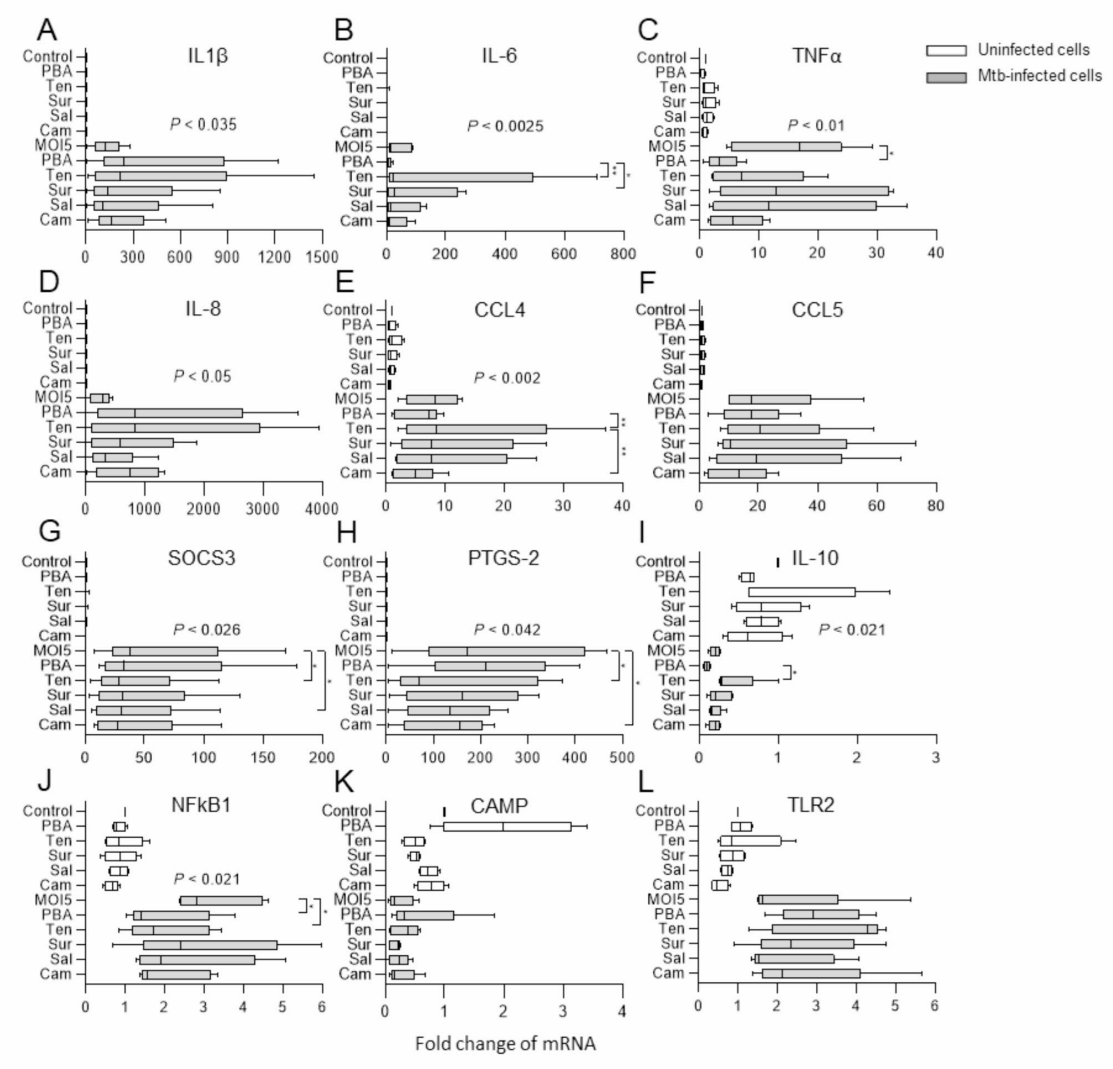
Multiplex RNA profiling of Mtb-infected macrophages treated with PBA or selected sirtuin inhibitors. Uninfected (white bars) and H37Rv-infected hMDMs (MOI5) (grey bars) were treated with medium only (Control or MOI5), PBA, tenovin (Ten), suramin (Sur), salermide (Sal) or cambinol (Cam) and assessed for mRNA expression of (**A**) IL-1β, (**B**) IL-6, (**C**) TNFα, (**D**) IL-8 (CXCL8), (**E**) CCL4, (**F**) CCL5, (**G**) SOCS-3, (**H**) PTGS-2 (COX2), (**I**) IL-10, (**J**) NFκB, (**K**) CAMP (LL-37), and (**L**) TLR2. Data assessed with Taqman array cards at 24 h is presented in box and whiskers graphs (median and min/max) from *n* = 5 donors (*n* = 3–4 donors for uninfected groups) and was analyzed using a Friedman and Dunn´s multiple comparisons test (Mtb-infected groups only). mRNA fold change in uninfected and untreated cells was set to 1. mRNA levels that are not visible in uninfected cells (**A**, **B**, **D** and **H**) due to a large fold induction in matched Mtb-infected cells, were in the range of 0-10-fold as compared to untreated and uninfected cells. *P < 0.05, **P < 0.01.

To gain a better understanding of the mechanisms associated with intracellular growth reduction of Mtb in macrophages, we customized a mRNA expression assay using Taqman array cards, consisting of 23 genes related with macrophage inflammation and defense (Supplementary Data S1). Results from 12 selected markers in uninfected and Mtb-infected hMDMs in response to PBA or the sirtuin inhibitors are shown in Fig. 7. Mtb infection had a clear impact on the expression of different markers in the host cells (Fig. 7). The effect of Mtb infection itself and treatment with PBA or the different sirtuin inhibitors mainly involved pro-inflammatory cytokines and chemokines (Fig. 7A-F) but also regulators of inflammation and macrophage effector function (Fig. 7G-L). Accordingly, PBA and all sirtuin inhibitors enhanced both IL-1β and IL-8 expression compared to the MOI5 infection control (Fig. 7A and D). The relative enhancement of IL-1β and IL-8 was higher in presence of PBA or tenovin as compared to suramin, salermide or cambinol (Fig. 7A and D). Instead, PBA reduced expression of IL-6 and CCL4 that was enhanced by all the sirtuin inhibitors except for cambinol (Fig. 7B and E). Accordingly, there was a significant increase in IL-6 mRNA levels in Mtb-infected macrophages upon treatment with tenovin (*P* < 0.006) and suramin (*P* < 0.02) as compared to PBA (Fig. 7B). In addition, CCL4 mRNA was significantly increased by tenovin as compared to both PBA (*P* < 0.006) and cambinol (*P* < 0.003) (Fig. 7E). Furthermore, PBA significantly (*P* < 0.01) reduced TNFα expression in Mtb-infected hMDMs but also uninfected cells, while presence of the sirtuin inhibitors, primarily suramin and salermide, seemed to restore TNFα expression (Fig. 7C). Compared to the MOI5 infection control, tenovin, salermide and cambinol demonstrated a significantly (*P* < 0.02–0.03) reduced expression of Suppressor of cytokine signaling 3 (SOCS-3) and Prostaglandin-endoperoxide synthase 2 (PTGS2, encoding cyclooxygenase-2, COX-2) (Fig. 7G-H). Contrary, PBA-treated Mtb-infected hMDMs expressed significantly (*P* < 0.01) lower IL-10 levels as compared to tenovin-treated cells, although the overall fold change of IL-10 was small (Fig. 7I). Mtb infection induced NFκB that was significantly downregulated by both PBA (*P* < 0.01) and tenovin (*P* < 0.03) treatment (Fig. 7J). In line with our previous findings^18,19^, CAMP encoding LL-37, was up-regulated by PBA, primarily in uninfected cells but also in Mtb-infected cells, whereas the sirtuin inhibitors did not affect CAMP expression in this assay (Fig. 7K). TLR2 mRNA was also induced by Mtb infection, but the HDAC inhibitors had minor effects on TLR2 expression (Fig. 7L). Unexpectedly, we failed to observe induction of the autophagy proteins ATG7, ATG14 and Beclin-1 (Supplementary Data S1), which may imply that different MOIs, compound doses, or kinetics may be required to detect these autophagy genes. Moreover, mRNA of CCL2, NADPH oxidase (NOX2), inducible oxide synthase (NOS2), S100A9, Nuclear factor erythroid 2 (Nrf2), Stimulator of interferon genes (STING), triggering receptor expressed on myeloid cells 2 (TREM2), and NOD-, LRR- and pyrin domain-containing protein 3 (NLRP3), were all expressed at low levels similar to uninfected and untreated control cells (Supplementary Data S1). In addition, RIF + INH treatment had a very small effect on macrophage inflammation in comparison to the MOI5 infection control (Supplementary Data S1). In summary, the results from this RNA expression array suggested that Mtb infection has a major impact on macrophage inflammation that can be modulated by HDAC inhibitors, and that the sirtuin inhibitors may promote a different RNA expression profile as compared to PBA.

## Discussion

Development of new antibiotics and efficient combinations of drugs with potential to shorten the very long TB treatment is one of the major goals in TB research. In this study, we explored a panel of selected HDAC inhibitors for the efficacy to reduce intracellular Mtb growth in human host cells. The findings support a significant effect of class III HDAC inhibitors, the sirtuin inhibitors tenovin, suramin, salermide and cambinol, to reduce Mtb growth in macrophages 45–75% when used in the µM-range as compared to average 40% for 2 mM PBA. Around half of the tested compounds had low or modest effect to reduce Mtb growth, which was primarily associated to induction of host cell cytotoxity. The effect of the HDAC inhibitors was relatively higher in fully differentiated macrophages as compared to bulk PBMC cultures containing monocytes. None of the sirtuin inhibitors, except tenovin, showed ability to reduce growth of extracellular Mtb bacilli, supporting a primary effect on boosting macrophage responses. Additive effects were observed upon combination treatment using sub-inhibitory doses of RIF or INH together with the sirtuin inhibitors, primarily suramin. Multiplex RNA profiling disclosed that Mtb infection induced profound inflammation responses in host macrophages that can be differentially modulated by PBA and the sirtuin inhibitors. Altogether, these findings support a role for sirtuin inhibitors as adjunct HDT in TB, aiming to reduce intracellular Mtb growth and modulate macrophage inflammation.

Majority of the HDAC inhibitors that we tested in this study have not yet received clinical approval, however, several broad-spectrum HDAC inhibitors including vorinostat, belinostat, romidepsin, and panobinostat, have been approved for treatment of different malignancies^24,28^. These HDAC inhibitors are well known to affect cancer cell viability and enhance immune cell functions^24,29^, however, the mechanisms required to defeat persistent intracellular Mtb infection may be different from targeting cancer cells. Several studies have been conducted to explore the effects of different HDAC inhibitors on Mtb growth control in macrophages^30–34^. Similar to our results, modest effects on intracellular Mtb growth inhibition were observed upon treatment of infected THP-1 macrophages with valproic acid and vorinostat, while these HDAC inhibitors clearly enhanced the activity of RIF and INH^33^. Non-selective HDAC inhibitors including trichostatin A, reduced growth of Mtb in infected M1 or M2 macrophages by downregulation of inflammatory cytokine responses^31^. Likewise, a specific HDAC3 inhibitor, RGFP966, was found to reduce intracellular Mtb growth in both MDMs and alveolar macrophages by downregulation of IL-6 and TNFα, but upregulation of IL-1β production^32^. RGFP966 exhibited optimal activity in the µM-range with a similar potency as compared to 2.5 mM PBA^32^. While enhanced HDAC activity is generally associated with gene silencing, HDAC inhibition in Mtb-infected macrophages may also result in reduced gene expression of mediators such as matrix metalloproteases (MMPs) -1 and − 3, which drive immunopathology in TB^35^. Likewise, HDAC6 inhibition with tubastatin A in mice has been shown to reduce anti-inflammatory IL-10 expression, which resulted in enhanced TNFα, IL-12, and IFN-γ levels in the lung and a subsequent reduction of mycobacterial growth^34^.

Sirtuins are NAD+-dependent HDAC enzymes with multifaceted roles that targets different proteins involved in various biological processes including autophagy, inflammation and immune cell metabolism^36^. Recently, a comprehensive study mapping immune response pathways in Mtb-infected M0, M1, and M2 polarized macrophages, described that Sirt2 expression was gradually increased by virulent Mtb in infected macrophages^37^. Treatment of Mtb-infected macrophages with the Sirt1 and 2 inhibitor, selisistat, reduced intracellular Mtb growth and enhanced IL-2 production by co-cultured CD4+ T cells^37^. Contrary, treatment with romidepsin, belinostat, entinostat, or tubastatin, failed to induce IL-2 in CD4+ T cells, indicating that these HDAC inhibitors could not affect the ability of Mtb-infected macrophages to present antigens to CD4+ T cells^37^. The authors suggested that Sirt2 was involved in the regulation of autophagy via Mtb-induced epigenetic modifications in ATG5-associated histones that affected Mtb growth in different macrophages subsets^37^. ATG5 was not included in the Taqman array used in our study, as it was recently shown that ATG7 and ATG14 are key autophagy proteins required for restricting Mtb replication in human macrophages^38^. While we failed to detect upregulation of these autophagy genes in response to PBA or the sirtuin inhibitors, it is possible that different kinetics and other sets of autophagy genes are involved in the regulation of intracellular Mtb growth. We have previously shown that PBA + vitD can upregulate ATG5 and Beclin-1 as well as LC3 in Mtb-infected macrophages, leading to induction of autophagy and Mtb growth control in an LL-37-dependent manner^18,19^. The therapeutic potential of Sirt2 inhibition was also demonstrated recently in a murine model of TB, where the Sirt2 inhibitor AGK2 restricted intracellular growth of both drug-susceptible and resistant strains of Mtb and enhanced the efficacy of INH^39^. Elevated Sirt2 expression have been associated with impaired autophagy and hypo-inflammation in free fatty acid-exposed murine macrophages that can be reversed by treatment with the selective Sirt2 inhibitor AK-7^40^. Interestingly, unpublished findings suggest that inhibition of Sirt2 in host cells can lower the availability of iron to Mtb bacilli via transcriptional regulation of iron transport proteins, which results in reduced bacterial load^41^. In contrast to Sirt 2, other studies have found an anti-inflammatory role for Sirt1 in host immune defense that support TB control. Sirt1 has been shown to be downregulated in Mtb-infected macrophages in vitro and in experimental TB models as well as in PBMCs obtained from TB patients^42,43^. Sirt1-deficient THP-1 cells displayed elevated Mtb loads upon infection^42^and ablation of Sirt1 in murine myeloid cells resulted in elevated NFkB activation and increased transcriptional activation of proinflammatory target genes such as IL-6 and TNF-α^43,44^as well as disruption of oxidative energy metabolism^44^. Instead, activation of Sirt1 with resveratrol ameliorated TB-associated inflammation and lung tissue pathology and reduced CFU counts in the lungs of Mtb-infected mice^42,43^. Sirt1 activation appeared to contribute to control of intracellular Mtb growth by inducing autophagy and phagosome-lysosome fusion^42^. Other studies corroborate these finding and show that resveratrol diminished TNF-induced inflammation but increased autophagy in human cells^45^. These results suggest that specific inhibition of Sirt1 may result in reduced autophagy but enhanced inflammation that could be deleterious in TB control. However, Sirt1/2 inhibition using sirtinol^46^or salermide^47^has been reported to induce growth arrest and pro-survival autophagy in human cells, which highlights the complexity of sirtuin regulation. Of note, selisistat, which is a more potent and selective Sirt1 inhibitor compared with other Sirt1 inhibitors^48^, had an overall weak effect to reduce intracellular Mtb growth in macrophages, indicating the anti-mycobacterial effects induced in human host cells may be primarily related to inhibition of Sirt2^36^. However, while HDAC inhibitors can alter immune cell differentiation and function by direct interaction with HDAC enzymes, these compounds may also target other proteins involving non-epigenetic mechanisms.

None of the selected sirtuin inhibitors in this study are currently approved for clinical use, but majority of previous studies of these compounds are based on experimental models. Moreover, none of the selected sirtuin inhibitors except suramin, has previously been studied in the context of Mtb infection. Instead, there are interesting findings from other infections and immunological diseases that we can learn from. It has been demonstrated that tenovin-1 could inhibit dengue virus replication in THP-1 monocytes and prevent virus-induced cytopathic effects via selective inhibition of Sirt2^49^. These results indicated that tenovin ameliorated denge infection by affecting the infected host cell, as tenovin had no direct effects on the virus or viral entry into cells^49^. Another study showed that tenovin induced mRNA expression of IL-1β and IL-6 in astrocytes^50^, which is in accordance with our findings. However, in a more recent study, tenovin reduced collagen deposition and tissue fibrosis in diabetic rats on a high-fat diet by stimulating antioxidants and inhibiting inflammatory cytokines including IL-6, IL-1β, TNFα, but instead increasing IL-10^51^. Moreover, Sirt inhibition using salermide increased IL-6 mRNA and protein expression in human lung epithelial cells likely via activation of the Akt pathway, which suggested that Sirt proteins are involved in regulation of IL-6 signaling^52^. At high doses (10–250 µM), cambinol has been shown to be a powerful inhibitor of inflammatory and innate immune responses via blockade of phosphorylation-induced MAPK signaling and expression of cytokines in macrophages and DCs upon microbial stimuli including TNF, IL-1β, IL-6, IL-12p40, and IFN-γ^53^. But most importantly, cambinol protected LPS-treated mice from endotoxic and septic shock indicating a role of cambinol in pharmacological treatment of inflammatory diseases^53^. Finally, suramin is the only sirtuin inhibitor among the selected compounds in this study that has been studied in TB. Accordingly, suramin was shown to be a potent and selective inhibitor of the Mtb RecA protein^54^, while the effects of suramin on host immunity is more controversial. Suramin is a 100-year-old drug with antiparastic and antipurinergic properties that was shown already a long time ago to be able to alter phagolysomal formation in macrophages and to protect macrophages from destruction by virulent Listeria infection by improving the bacteriostatic potential of the macrophages^55^. However, the effects of suramin in vivo in mice infected with Listeria or *M. bovis* infection appear complex and involved a biphasic effect of suramin treatment with an increased bacterial resistance only upon 8 days pre-treatment with suramin prior challenge^56^. Altogether, these studies highlight that additional in vitro and in vivo research is required to better understand the specific and nonspecific effects induced by tenovin, suramin, salermide and cambinol.

We observed that among the selected sirtuin inhibitors, tenovin had the capacity to reduce both extracellular and intracellular Mtb growth, indicating direct anti-mycobacterial effects of tenovin. Similarly, it has been discovered that PBA possess direct anti-mycobacterial activities independently from the induction of antimicrobial and anti-inflammatory actions in human macrophages^57^. Whereas eukaryotes have several sirtuins in their genomes, bacteria encode either one or two sirtuins, but the knowledge about bacterial sirtuins is still limited^58^. Previous data support that deletion of the only sirtuin present in Mtb, regulated colony morphology, biofilm formation and immunogenicity of an H37Ra mutant stain^59^. Interestingly, overexpression of a sirtuin-like protein in *M. smegmatis* displayed a higher resistance to INH as compared to the controls^60^. Notably, while suramin and tenovin showed synergistic or additive effects together with INH, no such effect could be detected upon combination treatment with INH and salermide or cambinol. Thus, it is possible that tenovin has an effect also to block mycobacterial sirtuins.

Implementation of adjunct HDTs is based on the ability of immunomodulatory compounds to support antibiotic treatment either by enhancing specific immune defense mechanisms, or inhibition of immunosuppressive pathways or reducing immunopathology. Appropriate levels of inflammatory cytokines and chemokines are required to clear Mtb bacteria at the local site of infection, while excessive and persistent cytokine/chemokine expression will contribute to pathological inflammation and tissue destruction in the lung^61,62^. We observed that Mtb infection had a great impact on early macrophage inflammation including cytokines and mediators which play important roles in innate immune activation and protection against Mtb and that can be modulated by the HDAC inhibitors. Primarily, the sirtuin inhibitors appeared to shift the early pro-inflammatory response in Mtb-infected macrophages to a variable extent involving relatively elevated levels of IL-6 and CCL4 as well as restored levels of TNFα, but reduced levels of IL-1β, IL-8 (except for tenovin), SOCS-3 and COX-2 as compared to PBA. This may suggest early M1 macrophage polarization towards a bactericidal state^8^. Chemokines including CCL3, CCL4, CCL5, and IL-8 induce activation and maturation of macrophages and are crucial for early intracellular restriction of Mtb growth, even though numerous reports also describe these chemokines to be elevated in patients with active TB disease^63^. A balanced TNFα response has been shown to be important in immune control of Mtb, as deficient levels will allow bacteria to replicate, while excess levels will enhance immunopathology^64,65^. Likewise, IL-1 and IL-6 contributes to host resistance by induction of early protective responses in the lung including both immune cell activation and recruitment^61^. Whereas IL-6 is mostly recognized for its pro-inflammatory activities and correlate for TB disease progression in the chronic phase^66^, it has been shown that IL-6 is essential for induction of bactericidal properties^67^but can also reinforce immunpolarization of anti-inflammatory M2 macrophages^68^. These findings suggest that ubiquitous IL-6, can enhance both M1 and M2 macrophage phenotypes displaying antimicrobial as well as immunoregulatory functions necessary for resolution of inflammation. This is relevant as the majority of HDT regimens in TB have so far been focused to reduce inflammation and resolve immunopathology including already approved non-steroidal anti-inflammatory drugs (NSAIDs) such as COX inhibitors^4^. Interestingly, data from a recent clinical trial disclosed that COX-2 inhibition with etoricoxib, resulted in enhanced intracellular growth of mycobacteria as compared to the control^69^. Lack of mycobacterial growth control in macrophages treated with the COX-2 inhibitor was associated with a lower RNA expression of TNF, IL-1β, CCL4, CXCL9, and CXCL10 and lower production of IFNβ and S100A8/A9 than the controls^69^. Contrary, in vitro treatment of Mtb-infected hMDM with the anti-inflammatory drug sulfasalazine reduced Mtb-induced COX-2 expression, which resulted in suppressed NFκB activation and production of IL-1β, TNFα and IL-6 that correlated to control of Mtb^70^. These reports highlight the importance of balancing HDTs to include immunomodulatory drugs that can achieve a state of immune equilibrium by both enhancing specific antimicrobial immunity and at the same time avoid overt inflammation. While our RNA array provided pilot data on early macrophage inflammation in response to PBA and the selected sirtuin inhibitors, additional in-depth studies would be required to confirm and extend on these findings. As such, chromatin accessibility assays and RNA sequencing will continue to address the genes or gene response modules that are differentially regulated by the sirtuin inhibitors in Mtb-infected macrophages at different doses and time-points. Additionally, studies elucidating the effects of the different sirtuin inhibitors on macrophage polarization and function as well as the effects on T cell responses will enhance the understanding for the potential of these compounds to control TB disease.

Phenotypic screening of compounds using high-content imaging have been established as a useful technological platform for cell-based assessment of intracellular Mtb growth in high-containment BSL-3 facilities^71,72^. The advantage of this method is rapid medium- to high-throughput analyses of compound libraries using multiple donors and conditions simultaneously including automated collection of images at regular intervals during the experiments. In addition, IncuCyte allows for assessment of morphological changes of host cells including evaluation of cell viability. It has been shown that there is a strong correlation between quantification of fluorescent bacteria inside cells and standard CFU counts^72^. Using primary cells from human donors generated a large donor-to-donor variation including differences in the relative growth rates of Mtb inside in vitro differentiated macrophages and sometimes also large inter-experimental variations. This may also be partly explained by different responsiveness of host cells to the HDAC inhibitors, similar to what has previously been observed regarding responsiveness of macrophages to BCG vaccination of healthy subjects^73^. Here, it was determined that enhanced anti-mycobacterial activity and IL1β response of Mtb-infected macrophages was detected in a sub-group of BCG-vaccinated individuals that also involved changes in global DNA methylation patterns in PBMCs from such BCG-responders compared to non-responders^74^. Future studies on the in vitro and in vivo effects of different sirtuin inhibitors on host immune responses in the lung will add to the understanding of how these compounds can be exploited for HDT in different forms of TB disease.

## Methods

### Ethics statement

All methods were carried out in accordance with relevant guidelines and regulations and informed consent was obtained from all subjects. Buffy-coat blood was obtained from healthy adult volunteers at the Karolinska Hospital Blood Bank, Stockholm, Sweden, after informed consent. Ethical approval for studies of immune cells in human blood from healthy donors was previously obtained from the Ethical Review Board in Stockholm, Sweden (2010/603 − 31/4).

All experiments involving risk groups 3 Mtb strains were performed according to the local guidelines at the biosafety level 3 (BSL-3) laboratories at Karolinska Institutet Campus Flemingsberg or at Linköpings University, after approval by the Swedish Work Environment Authority. All experimental protocols were approved by Karolinska Institutet, the Ethical Review Board in Stockholm, Sweden, and/or the Swedish Work Environment Authority.

### Human monocyte-derived macrophages (hMDMs)

Monocytes were isolated from peripheral blood obtained from healthy donors, using Lymphoprep™ (Alere technologies, Norway), involving separation by a density gradient followed by multiple centrifugation and wash steps. Peripheral blood mononuclear cells (PBMCs) were allowed to adhere in T75 culture flasks for 2–3 h at 37 °C in serum-free RPMI 1640 media (VWR, Radnor, PA). The non-adherent cells were removed by washing with phosphate buffered saline (PBS) and macrophages were differentiated from monocytes by culture for 7 days in RPMI media supplemented with 10% fetal calf sera (FCS), 2 mM L-glutamine, 1mM sodium pyruvate and 5mM HEPES, containing 50 ng/ml human macrophage colony-stimulating factor (M-CSF) (Life technologies, Carlsbad, CA). Human monocyte-derived macrophages (hMDMs) were detached using PBS buffer supplemented with 2.5% FCS and 2 mM EDTA at least 30 min at 37⁰C, counted and re-seeded in cell culture plates as described below.

## Mtb cultures

In this study, the laboratory strains, H37Ra (avirulent) or H37Rv (virulent) (ATCC 27294, Rockville, MD) expressing green fluorescent protein (GFP) (expression vector pFPV2) were used for infection of hMDMs. H37Ra- or H37Rv-GFP cultures were grown in complete TB medium (Middlebrook 7H9 media supplemented with 10% OADC (Oleic Albumin Dextrose Catalase) enrichment, Tween-80 (0.05%) and kanamycin (20 µg/ml) (Karolinska University Hospital, Stockholm, Sweden) for 2 weeks at 37 °C, before passage and sub-culture of bacteria for another week. Next, bacterial suspensions were pelleted and washed with PBS-Tween-80 (0.05%) twice and homogenized using pulse-sonication for 2 × 5 min. Bacterial aliquots (1 ml/cryotube) were adjusted to an optical density (OD) of 1 measured at 600 nm and frozen at -80 °C in complete TB medium containing 25% glycerol.

## Mtb infection model

On the day of the experiments, the required bacterial aliquots were thawed, washed, and pulse-sonicated before measurement of final bacterial concentration (OD = 0.2–0.3) and immediate infection of hMDMs or bulk PBMCs at a multiplicity of infection (MOI) of 1. This protocol was established to reduce intra-experimental variations using different batches of sub-cultured Mtb bacteria. For infection, bacteria were prepared in RPMI complete media without antibiotics. Briefly, hMDMs (10.000 or 4.000 cells/well, respectively) or PBMCs (50.000 or 20.000 cells/well, respectively) were infected with Mtb in 96- or 384-well plates at 37°C and MOI1. For PBMCs infection, the proportion of CD14+ monocytes were first determined with flow cytometry and thereafter the MOI was adjusted according to the absolute number of monocytes in bulk PBMC cultures. PBMC samples were stained with CD14-BV570 (Biolegend, San Diego, CA) and acquired on a BD LSRFortessa (BD Biosciences, Franklin Lakes, NJ). For hMDMs infection, the cells in 96-well plates were washed after 4 h of Mtb infection, while cells in 384-well plates were not washed after infection. For PBMCs infection, cells and bacteria were added at the same time without any washing step. Following Mtb infection, test compounds or positive and negative treatment controls were added to the Mtb-infected cell cultures for 24 h up to 3 or 5 days, before analyses of bacterial growth, host cell viability or mRNA expression of host cell markers. Z prime factor (Z’-factor) was used to determine the robustness of the assay using the means and standard deviation of the negative (MOI infection control in 0.5% DMSO) and positive (RIF + INH) controls, respectively.

### Commerical compounds

Commercial HDAC inhibitors compounds were either obtained from Sigma-Aldrich (Saint Louis, MO) or Selleckchem (Houston, TX), except for Sodium Phenylbutyrate (PBA) that was obtained from Cayman Chemical (Ann Arbor, MI). RIF and INH were obtained from Sigma Aldrich. The stock solutions of the compounds were prepared in 100% DMSO (Sigma-Aldrich) ranging from 50 mM to 10 mM depending upon their solubility. The compounds were then further diluted in complete RPMI media resulting in 0.1% DMSO concentration in the final solution. Likewise, the negative medium control also contained 0.1% DMSO. Mtb-infected macrophages were treated with the commercial HDAC inhibitors in the micromolar-range (0.0001-10 µM), while fixed doses were used of the internal HDAC inhibitor control PBA (2 mM) or the positive antibiotics control including a combination of RIF and INH (1 µg/ml). All compounds were used after careful titrations of the different doses to maximize efficacy and minimize toxicity.

### IncuCyte live-cell imaging

Intracellular Mtb growth in hMDMs was routinely assessed in real-time using the IncuCyte^®^S3 Live-Cell Analysis System (Sartorius, Göttingen, Germany). Automated IncuCyte microscopy quantified macrophage viability with the IncuCyte Cytotox Red Dye (Sartorius) and extra- as well as intracellular growth of H37Ra- or H37Rv-GFP upon incubation of cell cultures in the described test conditions for up to 5 days. At day 0, the cells were stained with Cytotox Red (250 nM/well) in complete medium and the positive control for cell death was cells fixed with 4% formaldehyde (Sigma). For assessment of extracellular Mtb growth, bacterial cultures containing 1 × 10^4^ mycobacteria/well were incubated with RIF + INH or the test compounds in the absence of hMDMs.

Upon image acquisition and analysis, 9 images/well in 96-well plate and 2 images/well in 384-well plate system at 20x magnification were captured at 12 h interval from day 0 until day 5. Fluorescent objects (green colour for bacteria and red colour for dying cells) were identified with the inbuilt IncuCyte S3 software enabling segmentation and background correction. Macrophage viability was quantified using red objects count per image. The number of dead cells subtracted from the total number of cells per image is equal to the number of viable cells which is then divided by the total number of cells per image and multiplied by 100 to determine the cell viability in percentage. Bacterial growth was quantified using the total integrated intensity (GCU x µm^2^/image). Data obtained from the software was exported to Excel and GraphPad Prism 9 (version 9.5.1) for analysis.

### Colony forming unit (CFU) assay

At day 3, Mtb-infected macrophages were lysed with 0.036% SDS-lysis buffer (Sodium dodecyl sulphate diluted in Milli-Q water) for 5 min to release intracellular bacteria and monitor growth on Middlebrook 7H10 agar plates (Karolinska University Hospital). Standard CFU counts were determined by diluting samples 1/10 to 1/10.000 by mixing 100 µl of bacterial suspension in 900 µl PBS and finally plating a volume of 200 µl on Middlebrook 7H10 agar plates with 10% OADC growth supplement. Each dilution was performed in duplicates, which showed a variation of less than 10%. Colonies were manually counted after incubation of the plates at 37 °C for a minimum of 21 days and CFU/ml was determined.

### TaqMan gene expression array cards

RNA expression analyses of macrophage inflammation included customized Taqman array cards (Thermo Fisher Scientific, Waltham, MA), allowing for mRNA quantification of 23 different genes in hMDM cultures. The customized Taqman array cards in 384-well format included the following genes: *IL1β*,* IL-6*,* TNF-α*,* IL-10*,* IL-8*,* CCL2*,* CCL4*,* CCL5*,* TLR2*,* Atg 7*,* Atg14*,* Beclin-1*,* CAMP (LL-37)*,* S100A9*,* NOS2 (iNOS)*,* CYBB (NOX2)*,* PTGS2 (COX2)*,* NFkB*,* SOCS-3*,* NLPR3*,* Nrf2*, *STING*, *TREM2*. mRNA expression in uninfected and H37Rv-infected hMDMs (MOI5) was assessed after 24 h incubation of cells in the described test conditions (PBA 2mM, tenovin, salermide and cambinol 10 µM, and suramin 1 µM). RNA was extracted from hMDMs using Ribopure RNA extraction kit as described by the manufacturer (Ambion, Thermo Fisher Scientific). cDNA was synthesized using Taqman Fast Advanced Mix and SuperScript IV VILO Master Mix from Thermo Fisher Scientific. Transcripts of the 23 selected inflammation genes, and also the housekeeping 18 S rRNA (reference gene), were measured in triplicates from the cDNA samples using quantitative real-time PCR (384-well format) with Applied Biosystems Instrument (ABI) 7900 HT with TLDA block (Applied Biosystems, Waltham, MA) at the Bioinformatics and Expression Analysis core facility (BEA) at Karolinska Institutet Campus Flemingsberg. Cycle threshold (Ct) values for the target genes were normalized to the Ct value for the housekeeping gene 18 S. The results were analysed by using the relative standard method. Briefly, the relative expression of the target genes was calculated by relating the Ct value for treated Mtb-infected macrophages to untreated Mtb-infected macrophages. Data are presented as fold change of mRNA.

### Statistical analyses

Values from two or more individual experiments including 3–12 donors in total, are presented as median in dot plot graphs showing individual data points or as median and range or interquartile range in bar graphs. Normally distributed data are presented as mean and standard error. Extra- and intracellular growth of Mtb is presented as percent (%) Mtb growth in hMDMs (or PBMCs) = (treatment condition) / (MOI1 infection control) x 100. The untreated MOI1 infection control corresponds to 100% growth, while the untreated and uninfected control corresponds to 0% growth. The statistical analyses used to calculate indicated *P*-values were conducted in GraphPad Prism 9 and included either a repeated measures (RM) ANOVA (normally distributed data) or Kruskal-Wallis and Dunn’s multiple comparisons test or Friedman’s test for > two groups, or a Mann-Whitney U-test for unpaired comparisons of two groups (non-normal distributed data). A *P*-value < 0.05* was considered statistically significant as indicated in the figures and text.

## Electronic supplementary material

Below is the link to the electronic supplementary material.

Supplementary Material 1

## Data Availability

RNA array datasets (Ct values and fold induction) of the 23 tested genes from five donors that were generated and analysed during the current study are available in the online Supplementary Data S1. All other data that supports the findings of this study are available from the corresponding author upon reasonable request.
